# Association of the Lactate/Albumin Ratio with Mortality and Hypovolemia in Critically Ill Patients: A Retrospective Cohort Study

**DOI:** 10.3390/jcm14176321

**Published:** 2025-09-07

**Authors:** Jakub Droś, Rafał Świstek, Patryk Kasongo, Jakub Konieczyński, Piotr Bielański, Agnieszka Sajdyk, Anna Wrzosek, Tomasz Składzień, Rafał Depukat, Maria Marusińska, Klaudia Czech, Katarzyna Frączek, Katarzyna Paciorek, Weronika Skoczeń, Bartłomiej Stachera, Weronika Chaba, Agata Peszek, Gabriela Pabian, Małgorzata Pawlik, Klaudia Zięba, Katarzyna Wolak, Anna Włodarczyk, Weronika Tomasiczek, Tomasz Drygalski, Michał Terlecki

**Affiliations:** 1 Department of Interdisciplinary Intensive Care, Jagiellonian University Medical College, 30-688 Krakow, Poland; jakub.dros@gmail.com (J.D.); rswistek@gmail.com (R.Ś.); patrykkasongo@gmail.com (P.K.); konieczynski.jakub@gmail.com (J.K.); bielanp@gmail.com (P.B.); agnieszkasajdyk@gmail.com (A.S.); a.wrzosek@uj.edu.pl (A.W.); t.skladzien@gmail.com (T.S.); depukat.rafal@gmail.com (R.D.); dtomec@gmail.com (T.D.); 2 Student’s Scientific Group in the Department of Interdisciplinary Intensive Care, Jagiellonian University Medical College, 30-688 Krakow, Poland; maria.marusinska@student.uj.edu.pl (M.M.); klaudia2.czech@student.uj.edu.pl (K.C.); katarzyna.fraczek@student.uj.edu.pl (K.F.); kasia.paciorek@student.uj.edu.pl (K.P.); weronika.skoczen@student.uj.edu.pl (W.S.); bartek.stachera@student.uj.edu.pl (B.S.); weronikachaba01@gmail.com (W.C.); gaga.pluta@gmail.com (A.P.); gabi.pabian@student.uj.edu.pl (G.P.); gosia.pawlik@student.uj.edu.pl (M.P.); claudia.zieba@student.uj.edu.pl (K.Z.); katarzyna.a.wolak@student.uj.edu.pl (K.W.); awlodarczykresearch@poczta.onet.pl (A.W.); weronika.tomasiczek@student.uj.edu.pl (W.T.)

**Keywords:** lactate, albumin, lactate/albumin ratio, hypovolemia, mortality, continuous renal replacement therapy

## Abstract

**Background/Objectives:** Previous research has demonstrated that the lactate/albumin ratio (L/A) may predict mortality among critically ill patients. Based on pathophysiological rationale, L/A may also correlate with volume status, however such an association has not been investigated extensively. This retrospective cohort study aimed to confirm the prognostic value of L/A and to assess the prognostic value of L/A and its relationship with hypovolemia severity in intensive care unit (ICU) patients. **Methods**: We analyzed data from consecutive adult patients admitted to the ICU. Admission L/A was evaluated in relation to 30-day mortality and indirect markers of volume status (mean arterial pressure on admission, median dose of norepinephrine and fluid intake within the first 24 h of ICU stay). **Results**: A total of 1421 patients were included. L/A ≥ 0.06 (estimated on the basis of ROC curve using the Youden index) was an independent predictor of 30-day mortality (HR = 1.423; 95%CI 1.183–1.712; *p* < 0.001). L/A moderately correlated with markers of absolute or relative hypovolemia, i.e., lower mean arterial pressure (r = −0.353, *p* < 0.001) on admission, higher norepinephrine dose (r = 0.506, *p* < 0.001) and greater fluid intake (r = 0.233, *p* < 0.001) within the first 24 h of ICU stay. Furthermore, L/A ≥ 0.06 on admission was an independent risk factor for the implementation of continuous renal replacement therapy (OR = 2.134; 95%CI 1.652–2.757; *p* = 0.001). **Conclusions**: L/A is not only a predictor of poor prognosis but also may be a valuable indirect marker of the extent of hypovolemia in critically ill patients. Further prospective studies are necessary to assess if this parameter should incline a decision for more aggressive fluid management in hypovolemic patients.

## 1. Introduction

Numerous studies have unequivocally demonstrated that elevated serum lactate level is an indirect determinant of tissue perfusion and are associated with an increased risk of mortality in critically ill patients [1,2]. These associations have been proven in many clinical scenarios such as sepsis, trauma, acute cardiac conditions or stroke [3,4,5,6]. Similarly, serum albumin is also considered a prognostic factor for increased mortality reflecting both systemic inflammation and chronic disease burden [7,8,9].

Since elevated lactate and decreased albumin levels are independent predictors of poor outcomes, clinical observations suggest that their combination may have greater prognostic value. Several studies have shown that the lactate/albumin ratio (L/A) predicts mortality among critically ill patients, however most reports have focused on specific subgroups of patients. Despite this evidence, L/A is not yet routinely applied in clinical practice, and its utility in a general ICU population still requires further validation. Importantly, beyond single-center and subgroup analyses, signals for the prognostic value of L/A have also emerged from broader settings (e.g., multicenter database cohorts and systematic reviews/meta-analyses across ICU populations). This strengthens the biological plausibility while underscoring the need to test L/A in unselected ICU admissions [10,11,12,13,14,15,16].

Additionally, absolute or relative hypovolemia remains one of the most frequent challenges in critical care. Therefore, many researchers still focus on studying factors enabling more appropriate fluid management [17,18]. In hypovolemia, reduced intravascular volume leads to inadequate tissue perfusion, forcing cells into anaerobic metabolism and increasing blood lactate levels—a marker of tissue hypoxia. This aligns with well-established pathophysiology of hypovolemic shock, whereby lactic acidosis reflects worsening perfusion and oxygen deficit. Concurrently, hypoalbuminemia may exacerbate the effects of hypovolemia: low albumin decreases plasma oncotic pressure, promoting further fluid loss into interstitial spaces and impairing circulatory volume—thus compounding hypoperfusion and raising the lactate/albumin ratio. In addition, inflammation-driven capillary leak and hemodilution can further lower albumin concentrations, amplifying the oncotic deficit and worsening effective circulating volume [19,20]. Thus, an elevated lactate/albumin ratio (reflecting both rising lactate due to poor perfusion and falling albumin from capillary leak or inflammation) may serve as a composite indicator of the severity of hypovolemia and tissue hypoxia, offering more prognostic value than lactate or albumin alone, however such correlation has not been investigated extensively.

The aim of this retrospective cohort study was to confirm the prognostic value of admission L/A for 30-day mortality in unselected cohort of critically ill patients admitted to the ICU and evaluate its relationship with indirect markers of absolute/relative hypovolemia (mean arterial pressure on admission, norepinephrine dose within 24 h, and fluid intake).

## 2. Materials and Methods

### 2.1. Data Collection and Study Endpoint

**Figure 1:**
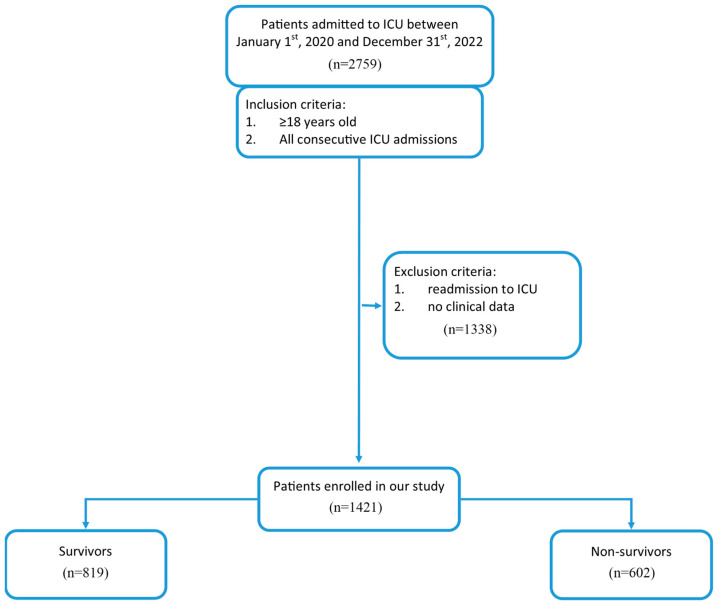
Flow chart of the study group.

This retrospective single-center study was conducted at the Jagiellonian University Medical College in Kraków, Poland. We included consecutive adult patients admitted to the intensive care unit (ICU) of the University Hospital in Kraków between January 2020 and December 2022, regardless of the reason for admission. The study was designed and reported in accordance with the STROBE (Strengthening the Reporting of Observational Studies in Epidemiology) guidelines for observational cohort studies (Supplementary Materials, Table S1) [21]. Eligible patients had both serum lactate and albumin levels measured immediately upon ICU admission. The study flowchart is presented in Figure 1. Clinical data—including demographics, medical history, inpatient clinical course, laboratory results, treatments, and in-hospital outcomes—were obtained from the electronic medical records of the University Hospital in Kraków. The Sequential Organ Failure Assessment (SOFA) score was calculated for each patient using data from the first 24 h of ICU stay. The primary endpoint was all-cause death within 30 days of ICU admission, ascertained via the National Electronic Population Registration System.

L/A was calculated for each patient included in our study by dividing serum lactate concentration [mmol/L] by serum albumin concentration [mg/mL], both measured at ICU admission. Lactate was measured using a point-of-care ICU blood gas analyzer (ABL90 FLEX, Radiometer Medical ApS, Copenhagen, Denmark) and albumin was measured in the hospital’s Central Diagnostic Laboratory. We used the first post-admission laboratory results, typically samples taken within 30 min of ICU arrival.

Additional parameters assessed on admission included mean arterial pressure [mmHg] and heart rate [bpm]. Within the first 24 h of ICU stay, we also recorded the total fluid intake per kilogram of body weight (mL/kg) and the median dose of norepinephrine (µg/kg/min). Total fluid intake included fluids contained in medications, regardless of route, as well as fluids from enteral nutrition and oral intake. Patients who died within 24 h of admission were excluded from analyses involving fluid intake.

Cases with missing data for any primary variables were excluded from the corresponding analyses to ensure the accuracy and reliability of the results; however, since the proportion of missing data was minimal (<5%), we considered the potential impact on the overall findings to be negligible and therefore did not apply any data imputation methods.

### 2.2. Outcome Measures

The primary outcome of the study was the prognostic value of L/A for 30-day all-cause mortality. Additionally, we assessed correlations between L/A and indirect markers of hypovolemia: mean arterial pressure on admission, total fluid intake per kilogram of body weight, and the median dose of norepinephrine administered during the first 24 h of ICU stay. The secondary outcome was the association between L/A and the initiation for continuous renal replacement therapy (CRRT) during ICU stay.

### 2.3. Statistical Analysis

Continuous values were presented as means with standard deviations (SD) or medians with interquartile ranges (IQR), depending on their distribution. Categorical variables were expressed as counts and percentages. Group comparisons were performed using the chi-squared test (with or without Yates’ correction, as appropriate) for categorical variables, and Student’s *t*-test or the Mann–Whitney U test for continuous variables, as appropriate. The prognostic performance of serum lactate, albumin and L/A for 30-day all-cause mortality was evaluated using receiver operating characteristic (ROC) curve analyses. Areas under the curve (AUCs) with 95% confidence intervals (CIs) were calculated. The optimal cut-off value for L/A was determined using Youden’s index.

The independent association between L/A (≥0.06 vs. <0.06) and 30-day mortality was assessed using multivariable Cox proportional hazards regression, adjusted for age and SOFA score as they demonstrated the strongest association with mortality. An alternative multivariable Cox regression model incorporating additional potential confounders such as sepsis and comorbidities was also prepared. Correlations between L/A and indirect indicators of hypovolemia (mean arterial pressure, fluid intake per kilogram, norepinephrine dose) were analyzed using Spearman’s rank correlation coefficients. The relationship between L/A and the requirement for continuous renal replacement therapy (CRRT) was examined using multivariable logistic regression, adjusted for relevant confounders. Results were presented as adjusted hazard ratios (HRs) or odds ratios (ORs) with corresponding 95% CIs. Statistical significance was defined as a two-tailed *p*-value < 0.05. Statistical analyses were performed with the R version 3.4.4 (R Foundation for Statistical Computing, Vienna, Austria). G*Power software version 3.1.9.7 (Heinrich Heine University Düsseldorf, Düsseldorf, Germany) was used to perform a power analysis in order to verify that our sample was sufficient for the study. We confirmed that the sample size included in our study allowed a power of 80% with an alpha error of 5%.

The study was conducted following the guidelines of the Declaration of Helsinki and was approved by the Bioethics Committee of Jagiellonian University, decision number 118.0043.1.160.2024.

## 3. Results

### 3.1. Clinical Characteristics of the Study Population

A total of 1421 patients admitted to the ICU between January 2020 and December 2022 were included in our study, comprising 840 medical and 581 surgical cases. The mean SOFA score at ICU admission was 10.14 ± 3.77. Mean serum lactate and albumin levels on admission were 3.61 ± 4.06 mmol/L and 29.64 ± 7.03 mg/mL, respectively. The mean lactate/albumin ratio (L/A) was 0.14 ± 0.19. The 30-day all-cause mortality rate was 42.4% (*n* = 602). Non-survivors were significantly older and had more comorbidities compared with survivors. Detailed baseline characteristics stratified by 30-day survival status are summarized in Table 1.

### 3.2. Association Between L/A and 30-Day Mortality

**Figure 2:**
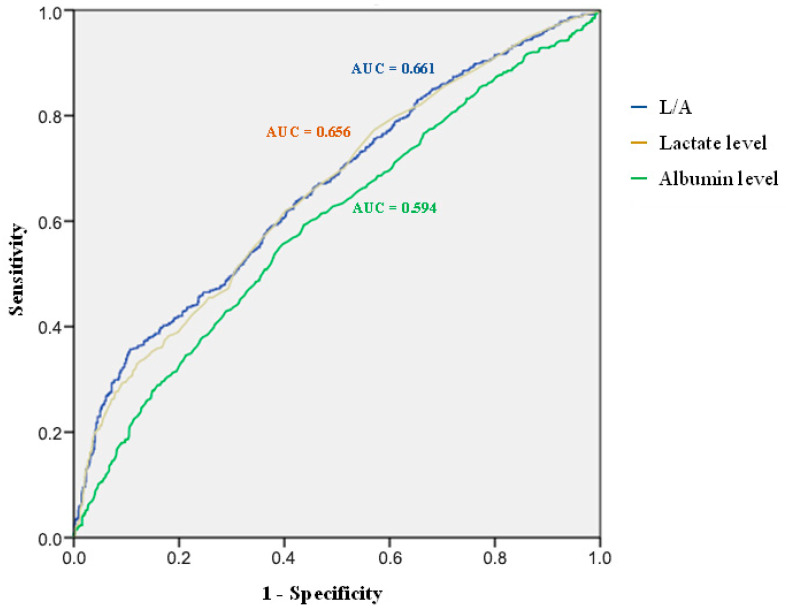
Receiver operating characteristic (ROC) curves for predicting 30-day mortality.

Lactate, albumin, and L/A were assessed for their predictive significance in 30-day all-cause mortality. Figure 2 displays receiver operating characteristic (ROC) curves. According to Table 2, the area under the curve (AUC) with 95% confidence intervals (CIs) shows that L/A (AUC: 0.661; 95% CI: 0.633–0.690) provided greater predictive performance than lactate alone (AUC: 0.656; 95% CI: 0.628–0.685) and albumin alone (AUC: 0.594; 95% CI: 0.564–0.624).

Using Youden’s index, the optimal cut-off for L/A was 0.06 (sensitivity 66.8% and specificity 53.6%). Based on this threshold, 632 patients (44.48%) had an L/A < 0.06 and 789 patients (55.52%) had L/A > 0.06. Compared with patients with L/A < 0.06, those with L/A > 0.06 exhibited worse clinical and laboratory parameters, longer ICU and hospital stays, more severe acid-base disturbance, and significantly higher SOFA score at admission (Table 3).

Furthermore, 30-day mortality was higher among patients with L/A ≥ 0.06. In a multivariable Cox regression model adjusted for age and SOFA score (Table 4), L/A ≥ 0.06 remained independently associated with 30-day mortality (HR: 1.423; 95% CI: 1.183–1.712; *p* < 0.001). The result for L/A is similar (HR: 1.587; 95% CI: 1.325–1.900; *p* < 0.001) while including in the multivariable analysis other potential confounders such as sepsis and comorbidities (Supplementary Materials, Table S2).

### 3.3. Correlation Between Hypovolemia Markers and L/A

**Figure 3:**
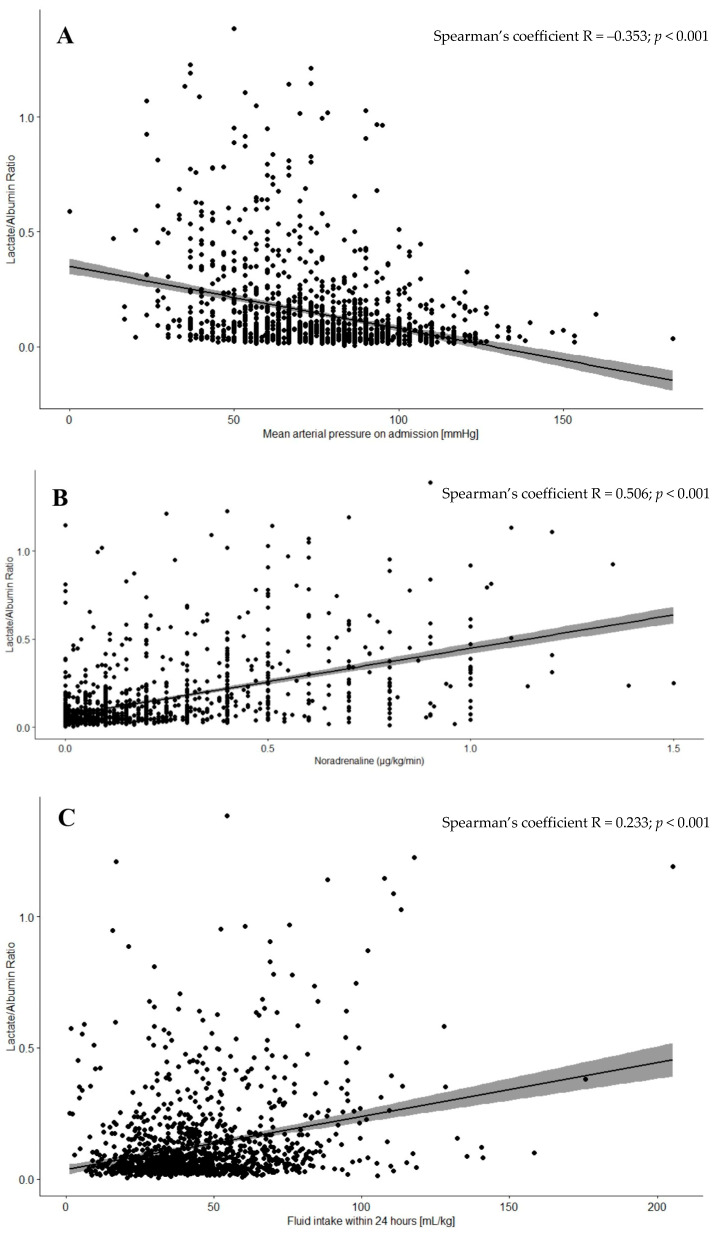
Correlation diagrams between L/A and mean arterial pressure on admission (**A**), dose of norepinephrine (**B**), and fluid within 24 h after admission (**C**).

Patients with L/A ≥ 0.06 required larger fluid volumes, received higher norepinephrine doses during the first 24 h, and had significantly lower mean arterial pressure upon ICU admission compared with those with L/A < 0.06 (Table 3). Additionally, correlation analysis showed that each of these measures of absolute or relative hypovolemia was moderately but statistically significantly correlated with greater L/A levels upon admission. Correlation charts are displayed in Figure 3.

### 3.4. Association Between L/A and CRRT Requirement

Continuous renal replacement therapy (CRRT) during ICU stay was required in 142 of 632 patients (22.5%) with L/A < 0.06 and in 337 of 789 patients (42.7%) with L/A ≥ 0.06; this difference was statistically significant (*p* < 0.001). In a multivariable logistic regression model adjusted for age, comorbidities, and admission category, L/A ≥ 0.06 was independently associated with greater odds of CRRT initiation (OR: 2.134; 95% CI: 1.652–2.757; *p* < 0.001) (Table 5).

## 4. Discussion

Our study evaluated predictive value of L/A for short-term mortality and its correlation with volume status. We demonstrated that L/A measured upon ICU admission a valuable indicator of worse outcomes. Moreover, L/A correlated with markers of absolute and relative hypovolemia, as reflected by hemodynamic parameters and treatment-related variables within the first 24 h of ICU stay.

Although previous reports confirmed the prognostic value of L/A, its application in routine clinical practice remains limited. For example, Wang et al. verified the utility of L/A in predicting 28-day mortality in a cohort of cases with acute respiratory distress syndrome while Jin et al. demonstrated it in patients with acute myocardial infarction [22,23]. Similarly to other authors, we also found that L/A had better predictive value compared to lactate or albumin levels considered separately [22,24,25]. Although these studies differ in terms of study population, adopted cut-off point or general mortality rate most of them promote the usefulness of L/A. However, as mentioned before, the majority of studies are limited to selected severe conditions, i.e., heart failure, septic shock, liver or kidney injury in contrast to our analysis of all consecutive, unselected cases with critical illness [11,12,13,14,15,16]. By including all patients referred to the intensive care unit regardless of admission diagnosis, we sought to develop a universal and easily applicable tool that could assist in patient stratification and guiding early therapeutic decisions. This issue has not been previously addressed in the literature, however it requires further validation and, subsequently, prospective studies that incorporate clinical management strategies.

Interestingly, in our study cases with higher L/A were characterized by significantly shorter ICU and hospital length of stay. While shorter length of stay usually indicates faster recovery, fewer complications, and more efficient use of critical care resources, in this context it likely reflects higher mortality in this group of patients.

Despite the pathophysiological rationale the correlation between L/A and surrogate markers of hypovolemia is a novel finding that has not been investigated in previous studies. Currently, significant efforts are directed toward identifying reliable tools and prognostic markers capable of accurately distinguishing between hypo- and hypervolemic cases [26,27]. Positive fluid balance has been consistently associated with adverse outcomes, including increased morbidity and mortality, in the majority of ICU populations. Nevertheless, a subset of patients with absolute or relative hypovolemia may still derive substantial benefit from timely and appropriately targeted fluid resuscitation. Developing a marker that can guide clinicians in tailoring fluid therapy—avoiding harmful fluid overload while ensuring adequate perfusion in hypovolemic states—remains a key challenge and a priority for improving critical care outcomes [28,29,30].

We focused on parameters such as mean arterial pressure, daily fluid intake or the median dose of norepinephrine that may have linked to the extent of hypovolemia as well as the severity of the condition resulting from other reasons. Although our results indicate a moderate degree of correlation, our findings should be considered valuable, because statistical significance was maintained even though the most severely ill patients (those who died before 24 h of ICU admission) were not included in this analysis. However, we are aware that in order to avoid misinterpretation further prospective research is required to verify whether L/A on ICU admission, beyond prognosis estimation, should determine more specific therapeutic decisions.

As a secondary outcome we revealed that higher L/A value observed on admission to the ICU was associated with an increased probability of CRRT implementation among critically ill patients. Only a few similar studies in the literature described the association between elevated L/A and the development of severe acute kidney injury or multiple-organ dysfunction syndrome in septic cases [20,31]. Other reports have linked elevated L/A to worse outcomes in patients with acute kidney injury or undergoing CRRT [32,33]. However, to our knowledge, no study has specifically investigated the ability of admission L/A to predict the need for subsequent CRRT use in the population of critically ill patients. We believe that our finding emphasizes the prognostic value of L/A in many aspects. Nevertheless, indications and timing of CRRT are still a matter of discussion, including sepsis severity, fluid overload, baseline creatinine or urine output, and may confound our results [34,35]. However, L/A might shed additional light on cases who are at risk for kidney failure requiring CRRT.

This study has several strengths. The large, unselected ICU cohort enrolled irrespective of admission indications increases generalizability across diverse critical care populations. Given the highly heterogeneous nature of critically ill patients—who often present with multiple organ failures—we aimed to identify a universal prognostic marker that could be rapidly calculated upon admission.

However, important limitations must be acknowledged. Although data were collected systematically, the possibility of selection, measurement, or confounding bias cannot be fully excluded. Efforts to mitigate these biases included consecutive enrollment of patients to reduce selection bias, reliance on standardized hospital laboratory systems to limit measurement bias, and adjustment for key confounders (age and SOFA score) in multivariable models to reduce confounding bias.

The retrospective design restricted causal inference and limited us to data recorded in medical records. Hypovolemia was assessed only through indirect surrogates that could be influenced by numerous confounders including cardiac output, vasoplegia or severity of disease. Baseline diagnoses were not considered in the analysis as many cases admitted to the ICU had a multifactorial cause of the disease, these potential factors were unmeasurable, and our aim was to assess L/A as a universal tool regardless of the reason for hospitalization.

Furthermore, although more advanced hemodynamic measures—such as passive leg raising, venous collapsibility index, transthoracic echocardiography, or parameters from transpulmonary thermodilution [36]—are routinely used in our unit, they were not consistently documented in all patients. An analysis of such data from a minority of our population would lack statistical value and could lead to erroneous conclusions. Future studies incorporating comprehensive hemodynamic monitoring should prioritize prospective validation and include direct assessments of volume status to validate the role of L/A as a marker of hypovolemia.

Additionally, the retrospective nature of the study necessitated the exclusion of patients who died within the first 24 h of ICU stay from the analysis of fluid status. This may have led to an underestimation of the association between L/A and early hypovolemia, which could potentially be stronger in this early fatality group.

No formal sensitivity analyses were performed. However, the predictive threshold of L/A was derived using the Youden index in ROC analysis, and its robustness was supported by consistent findings in multivariable models. Moreover, the relatively low specificity of the ROC-derived cutoff and a moderate AUC suggest that its clinical applicability may be limited. Clinical decisions should not be based solely on whether L/A ratio exceeds a reference threshold but must also take into account its absolute value as well as other patient-specific medical factors. Although our results are statistically significant, external validation in independent and geographically diverse ICU populations is essential to assess the reproducibility and clinical utility of the lactate/albumin ratio.

## 5. Conclusions

Our study results confirm the association between L/A and mortality risk in unselected critically ill patients. Moreover, L/A may potentially serve as a useful marker of the extent of absolute or relative hypovolemia pending confirmation through further studies using direct parameters of volume status. Additional research is required to determine whether L/A should support a decision for more aggressive fluid management in hypovolemic patients. Our study should motivate researchers to conduct future prospective studies on the potential of L/A in hemodynamic assessment and risk stratification that are necessary before it could be reliably used to guide therapy.

## Figures and Tables

**Table 1:** Baseline clinical characteristics according to 30-day survival status.

Parameter	Survivors (*n* = 819, 57.6%)	Non-Survivors (*n* = 602, 42.4%)	*p*-Value
Age [years], median (IQR)	62 (47; 70)	67 (55; 74)	<0.001
Male gender, *n* (%)	502 (61.3)	379 (63.0)	0.280
Body mass index, mean (SD) ^a^	27.79 (±6.33)	28.73 (±6.91)	0.090
Comorbidities			
Diabetes mellitus, *n* (%)	203 (24.8)	175 (29.1)	0.041
Arterial hypertension, *n* (%)	388 (47.4)	337 (56.0)	0.001
Obstructive pulmonary disease, *n* (%)	55 (6.7)	59 (9.8)	0.022
Ischemic heart disease, *n* (%)	108 (13.2)	132 (21.9)	<0.001
Chronic kidney disease, *n* (%)	94 (11.5)	88 (14.6)	0.048
Chronic liver disease, *n* (%)	21 (2.6)	37 (6.1)	0.001
Heart failure, *n* (%)	121 (14.8)	130 (21.6)	0.001
Active malignancy, *n* (%)	96 (11.7)	85 (14.1)	0.104
Previous stroke, *n* (%)	51 (6.2)	32 (5.3)	0.272
Admission category			
Medical, *n* (%)	454 (55.4)	386 (64.1)	0.001
Surgical, *n* (%)	365 (44.6)	216 (35.9)	0.001
Admission diagnosis category			
Respiratory, *n* (%)	238 (29.1)	194 (32.2)	0.281
Cardiovascular ^b^, *n* (%)	156 (19.0)	164 (27.2)	<0.001
Other medical, *n* (%)	14 (1.7)	6 (1.0)	0.260
Non-operative trauma, *n* (%)	46 (5.6)	22 (3.7)	0.087
Postoperative, *n* (%)	365 (44.6)	216 (35.9)	0.001

Abbreviations: IQR—interquartile range, SD—standard deviation. ^a^ data available in 1366 patients. ^b^ the cardiovascular admission category includes admissions due to cardiovascular failure or insufficiency from hypertensive crisis, rhythm disturbances, acute decompensation of heart failure, hemorrhagic/hypovolemic shock, sepsis and dissecting aortic aneurysm.

**Table 2:** The predictive value of L/A, lactate level and albumin level on 30-day mortality on the basis of Receiver Operating Characteristics (ROC) curves with 95% confidence intervals of calculated areas under the ROC curves.

Parameter	AUC	95%CI of AUC	*p*-Value
L/A	0.661	0.633–0.690	<0.001
Lactate level	0.656	0.628–0.685	<0.001
Albumin level	0.594	0.564–0.624	<0.001

Abbreviations: CI—confidence interval, AUC—area under the ROC curve.

**Table 3:** Clinical, laboratory and prognostic parameters on admission to the ICU according to L/A.

Parameter	L/A < 0.06 (*n* = 632, 44.48%)	L/A ≥ 0.06 (*n* = 789, 55.52%)	*p*-Value
SOFA score, mean (SD)	8.71 (±3.33)	11.3 (±3.70)	<0.001
ICU length of stay [days], mean (SD)	16.80 (±16.31)	14.14 (±15.92)	0.002
Hospital length of stay [days], mean (SD)	25.36 (±18.16)	21.17 (±18.66)	<0.001
Lactate level [mmol/L], median (IQR)	1.10 (0.90; 1.50)	3.80 (2.40; 7.00)	<0.001
Albumin level [mg/mL], median (IQR)	32.40 (28.20; 36.48)	27.30 (22.30; 32.30)	<0.001
L/A, median (IQR)	0.04 (0.28; 0.47)	0.13 (0.085; 0.258)	<0.001
Anion gap [mEq/L], median (IQR) ^1^	10.00 (7.80; 12.40)	12.90 (9.70; 16.55)	<0.001
Base excess [mmol/L], median (IQR) ^2^	−2.00 (−4.90; 1.20)	−6.50 (−11.00; −2.00)	<0.001
pH, median (IQR)	7.344 (7.28; 7.40)	7.270 (7.16; 7.35)	<0.001
Mean arterial pressure on admission [mmHg], median (IQR)	83.33 (71.67; 96.67)	70.00 (56.67; 86.67)	<0.001
Heart rate [bpm], median (IQR)	85 (75; 100)	95 (75; 115)	<0.001
Dose of norepinephrine within 24 h [µg/kg/min], median (IQR)	0.05 (0.00; 0.12)	0.20 (0.05; 0.40)	<0.001
Fluid intake within 24 h [mL/kg], median (IQR) ^3^	36.94 (27.70; 48.05)	43.91 (33.14; 62.21)	<0.001
CRRT during ICU stay, *n* (%)	142 (22.5)	337 (42.7)	<0.001
30-day mortality, *n* (%)	198 (31.3)	404 (51.2)	<0.001

Abbreviations: CRRT—continuous renal replacement therapy, ICU—intensive care unit, IQR—interquartile range, SD—standard deviation, SOFA—Sequential Organ Failure Assessment. ^1^ data available in 1401 patients. ^2^ data available in 1418 patients. ^3^ data available in 1350 patients (71 died within 24 h after ICU admission).

**Table 4:** The multivariable Cox regression model for predicting 30-day mortality.

Variable	HR (95%CI)	*p*-Value
Age (1 year)	1.010 (1.005–1.016)	<0.001
SOFA score (1 point)	1.148 (1.119–1.178)	<0.001
L/A ≥ 0.06 (0/1)	1.423 (1.183–1.712)	<0.001

Abbreviations: HR—hazard ratio, CI—confidence interval.

**Table 5:** Risk factors for the implementation of continuous renal replacement therapy in the multivariate logistic regression model.

Variable	OR (95%CI)	*p*-Value
Age (1 year)	0.993 (0.985–1.002)	0.115
Diabetes mellitus (0/1)	1.083 (0.807–1.454)	0.596
Arterial hypertension (0/1)	1.361 (1.027–1.805)	0.032
Obstructive pulmonary disease (0/1)	0.756 (0.481–1.189)	0.226
Ischemic heart disease (0/1)	1.089 (0.773–1.535)	0.625
Chronic kidney disease (0/1)	2.814 (1.966–4.029)	<0.001
Chronic liver disease (0/1)	1.974 (1.104–3.530)	0.022
Active malignancy (0/1)	1.191 (0.836–1.696)	0.333
Previous stroke (0/1)	0.807 (0.571–1.140)	0.224
Sepsis on admission (0/1)	2.940 (2.304–3.750)	<0.001
Surgical procedure before admission (0/1)	1.002 (0.779–1.289)	0.985
L/A ≥ 0.06 (0/1)	2.134 (1.652–2.757)	<0.001

Abbreviations: OR—odds ratio, CI—confidence interval.

## Data Availability

The data presented in this study are available on request from the corresponding author.

## Supplementary Materials

The following supporting information can be downloaded at: https://www.mdpi.com/article/10.3390/jcm14176321/s1, Table S1: STROBE Statement—Checklist of items that should be included in reports of cohort studies. Table S2: The alternative multivariable Cox regression model for predicting 30-day mortality adjusted for age, SOFA score, sepsis and comorbidities.

## Abbreviations

The following abbreviations are used in this manuscript:

AUC	Area Under the Curve
CI	Confidence Interval
CRRT	Continuous Renal Replacement Therapy
HR	Hazard Ratio
ICU	Intensive Care Unit
IQR	Interquartile Range
L/A	Lactate/Albumin Ratio
OR	Odds Ratio
ROC	Receiver Operating Characteristic
SD	Standard Deviation
SOFA	Sequential Organ Failure Assessment
STROBE	Strengthening the Reporting of Observational Studies in Epidemiology
