# Supplementary material for: Association of the Lactate/Albumin Ratio with Mortality and Hypovolemia in Critically Ill Patients: A Retrospective Cohort Study
Source: J Clin Med. 2025 Sep 7;14(17):6321. doi: 10.3390/jcm14176321 (PMC12429822; doi:10.3390/jcm14176321)
Supplement: Supplementary file 1 [file jcm-14-06321-s001.zip › Table S1. STROBE_checklist.pdf]

Table S1. STROBE Statement—Checklist of items that should be included in reports of ***cohort studies***.

	Item No	Recommendation	Lines in manuscript
**Title and abstract**	1	(*a*) Indicate the study's design with a commonly used term in the title or the abstract	Indicated in the title (lines 2–4).
		(*b*) Provide in the abstract an informative and balanced summary of what was done and what was found	The abstract includes a structured summary of the background, methods, results and conclusion (lines 14–35).
**Introduction**			
Background/rationale	2	Explain the scientific background and rationale for the investigation being reported	Provided in the Introduction (lines 41–73).
Objectives	3	State specific objectives, including any prespecified hypotheses	Last paragraph of the Introduction (lines 74–78).
**Methods**			
Study design	4	Present key elements of study design early in the paper	Described early in the Methods section (lines 80–113).
Setting	5	Describe the setting, locations, and relevant dates, including periods of recruitment, exposure, follow-up, and data collection	Study setting and time frame are detailed in lines 81–84.
Participants	6	(*a*) Give the eligibility criteria, and the sources and methods of selection of participants. Describe methods of follow-up	Inclusion/exclusion criteria and data source are detailed in lines 87–93.
		(*b*) For matched studies, give matching criteria and number of exposed and unexposed	Not applicable – no matching was performed.
Variables	7	Clearly define all outcomes, exposures, predictors, potential confounders, and effect modifiers. Give diagnostic criteria, if applicable	All variables (exposures, outcomes, confounders) are defined in lines 114–120.
Data sources/ measurement	8*	For each variable of interest, give sources of data and details of methods of assessment (measurement). Describe comparability of assessment methods if there is more than one group	Source and method of measurement of each variable are provided in lines 89–108.
Bias	9	Describe any efforts to address potential sources of bias	No formal bias analysis, discussed in limitation section.
Study size	10	Explain how the study size was arrived at	Explained in lines 145–146.
Quantitative variables	11	Explain how quantitative variables were handled in the analyses. If applicable, describe which groupings were chosen and why	Quantitative variables are described with methods for transformation and grouping (lines 122–130).

Statistical methods	12	(*a*) Describe all statistical methods, including those used to control for confounding	Statistical methods are described in lines 121–146.
		(*b*) Describe any methods used to examine subgroups and interactions	No subgroup or interaction analyses were performed.
		(*c*) Explain how missing data were addressed	Missing data handling is noted by footnotes under tables (lines 162, 186–188); no imputation was performed. Importantly, for all main parameters analysed – 100% of data were available and there were no missing data.
		(*d*) If applicable, explain how loss to follow-up was addressed	Not applicable – 30-day mortality was available for all patients from national registry
		(*e*) Describe any sensitivity analyses	Not done – discussed in limitation section
**Results**			
Participants	13*	(a) Report numbers of individuals at each stage of study—eg numbers potentially eligible, examined for eligibility, confirmed eligible, included in the study, completing follow-up, and analysed	Number of included patients and flowchart are presented in lines 152–153 and Figure 1.
		(b) Give reasons for non-participation at each stage	Reasons for exclusion are described based on predefined criteria.
		(c) Consider use of a flow diagram	A study flowchart (Figure 1) is included.
Descriptive data	14*	(a) Give characteristics of study participants (eg demographic, clinical, social) and information on exposures and potential confounders	Baseline characteristics and confounders are described in Table 1.
		(b) Indicate number of participants with missing data for each variable of interest	Missing data are indicated in table footnotes.
		(c) Summarise follow-up time (eg, average and total amount)	Follow-up time is fixed at 30 days; ICU/hospital stay durations are reported.
Outcome data	15*	Report numbers of outcome events or summary measures over time	Outcome data (mortality) are reported in lines 167–172), and in Tables 2 and 3.
Main results	16	(*a*) Give unadjusted estimates and, if applicable, confounder-adjusted estimates and their precision (eg, 95% confidence interval). Make clear which confounders were adjusted for and why they were included	Unadjusted and adjusted HRs with 95% CIs are provided in lines 190–195.

		(*b*) Report category boundaries when continuous variables were categorized	Categories for continuous variables are reported (e.g. age, SOFA).
		(*c*) If relevant, consider translating estimates of relative risk into absolute risk for a meaningful time period	Absolute risk was not calculated.
Other analyses	17	Report other analyses done—eg analyses of subgroups and interactions, and sensitivity analyses	Other analyse include ROC comparison.
**Discussion**			
Key results	18	Summarise key results with reference to study objectives	Key findings are summarized in the first paragraph of the Discussion (lines 221–225).
Limitations	19	Discuss limitations of the study, taking into account sources of potential bias or imprecision. Discuss both direction and magnitude of any potential bias	Study limitations are discussed in detail in the Limitations section (lines 283–314).
Interpretation	20	Give a cautious overall interpretation of results considering objectives, limitations, multiplicity of analyses, results from similar studies, and other relevant evidence	Interpretation considers objectives, existing literature, and clinical relevance (lines 226–282).
Generalisability	21	Discuss the generalisability (external validity) of the study results	Generalizability and need for external validation are addressed in the limitation section (lines 300–303, 314–316).
**Other information**			
Funding	22	Give the source of funding and the role of the funders for the present study and, if applicable, for the original study on which the present article is based	Funding statement included: 'This research received no external funding' (line 354).

*Give information separately for exposed and unexposed groups.

Note: An Explanation and Elaboration article discusses each checklist item and gives methodological background and published examples of transparent reporting. The STROBE checklist is best used in conjunction with this article (freely available on the Web sites of PLoS Medicine at http://www.plosmedicine.org/, Annals of Internal Medicine at http://www.annals.org/, and Epidemiology at http://www.epidem.com/). Information on the STROBE Initiative is available at http://www.strobe-statement.org.
