# Supplementary material for: Association of the Lactate/Albumin Ratio with Mortality and Hypovolemia in Critically Ill Patients: A Retrospective Cohort Study
Source: J Clin Med. 2025 Sep 7;14(17):6321. doi: 10.3390/jcm14176321 (PMC12429822; doi:10.3390/jcm14176321)
Supplement: Supplementary file 1 [file jcm-14-06321-s001.zip › Table S2. Cox_regression_model.pdf]

Table S2. The alternative multivariable Cox regression model for predicting 30-day mortality adjusted for age, SOFA score, sepsis and comorbidities.

Variable	HR (95%CI)	*P*-value
Age (1 year)	1.011 (1.006-1.019)	< 0.001
SOFA score (1 point)	1.090 (1.066-1.114)	< 0.001
Sepsis (0/1)	0.987 (0,795-1.227)	0.909
Diabetes mellitus (0/1)	0.936 (0.771-1.138)	0.508
Arterial hypertension (0/1)	1.015 (0.843-1.223)	0.874
Obstructive pulmonary disease (0/1)	1.107 (0.840-1.457)	0.470
Ischemic heart disease (0/1)	1.290 (1.030-1.616)	0.027
Chronic kidney disease (0/1)	0.925 (0.726-1.178)	0.527
Chronic liver disease (0/1)	1.482 (1.049-2.094)	0.025
Heart failure (0/1)	1.045 (0.836-1.306)	0.700
Active malignancy (0/1)	1.048 (0.828-1.328)	0.697
L/A ≥ 0.06 (0/1)	1.587 (1.325-1.900)	< 0.001

Abbreviations: HR – hazard ratio, CI – confidence interval.
